# Bilateral step length estimation using a single inertial measurement unit attached to the pelvis

**DOI:** 10.1186/1743-0003-9-9

**Published:** 2012-02-08

**Authors:** Alper Köse, Andrea Cereatti, Ugo Della Croce

**Affiliations:** 1 Biomedical Sciences Department, University of Sassari, Sassari, Italy

**Keywords:** Inertial measurement, Gait analysis, Gait parameters, Accelerometer, Step length, Gait monitoring, Stride length, Inertial sensor, Wearable.

## Abstract

**Background:**

The estimation of the spatio-temporal gait parameters is of primary importance in both physical activity monitoring and clinical contexts. A method for estimating step length bilaterally, during level walking, using a single inertial measurement unit (IMU) attached to the pelvis is proposed. In contrast to previous studies, based either on a simplified representation of the human gait mechanics or on a general linear regressive model, the proposed method estimates the step length directly from the integration of the acceleration along the direction of progression.

**Methods:**

The IMU was placed at pelvis level fixed to the subject's belt on the right side. The method was validated using measurements from a stereo-photogrammetric system as a gold standard on nine subjects walking ten laps along a closed loop track of about 25 m, varying their speed. For each loop, only the IMU data recorded in a 4 m long portion of the track included in the calibrated volume of the SP system, were used for the analysis. The method takes advantage of the cyclic nature of gait and it requires an accurate determination of the foot contact instances. A combination of a Kalman filter and of an optimally filtered direct and reverse integration applied to the IMU signals formed a single novel method (Kalman and Optimally filtered Step length Estimation - KOSE method). A correction of the IMU displacement due to the pelvic rotation occurring in gait was implemented to estimate the step length and the traversed distance.

**Results:**

The step length was estimated for all subjects with less than 3% error. Traversed distance was assessed with less than 2% error.

**Conclusions:**

The proposed method provided estimates of step length and traversed distance more accurate than any other method applied to measurements obtained from a single IMU that can be found in the literature. In healthy subjects, it is reasonable to expect that, errors in traversed distance estimation during daily monitoring activity would be of the same order of magnitude of those presented.

## Background

The measurement of temporal and spatial features of gait is essential for the assessment of gait abnormalities and the quantitative evaluation of treatment outcomes [1]. In particular, amplitude, variability and asymmetry of step length (SL) have been shown to be effective outcomes of walking ability. In fact, they are strongly related to the propulsion generation and can be representative of the compensatory mechanisms adopted in pathological walking [2,3]. Having access to instruments capable of gathering information about the patient walking ability outside the laboratory [4], during daily life with no space limitations and for prolonged periods of time is of paramount importance in numerous clinical applications [5].

Inertial measurement units (IMU) are strong candidates for these applications. Those IMUs, featuring 3-axis accelerometers and gyroscopes [6], can be employed to estimate the SL during walking. An estimate of the SL can be obtained by double integrating the IMU acceleration component in the direction of progression (DoP) between the instants of two consecutive heel strikes (HS).

However, the implementation of the procedure described above requires the solution of a number of critical issues:

a) the identification of the foot contact time instances (gait events);

b) the determination of the IMU orientation with respect to the GF [7-9];

c) the compensation for the drift affecting the accelerometer and gyroscope signals [10,11];

d) the estimation of the initial velocity values for the integration of the acceleration along the DoP [12].

The most straightforward solution to determine both right and left SL (*rSL *and *lSL*) would be to place an IMU on each foot so that velocity and the orientation of each IMU can be set to zero at the beginning of the integration interval [7,13,14].

However, when the focus is on the description of the individual motor capacity ("can do in daily environment") and performance ("does do in daily environment") [15], the requirement of a light, small and minimally obstructive setup is of primary importance. Therefore, it would be desirable to obtain the same information using a single, discomfort-free IMU.

To the authors' knowledge, all the studies in the literature, based upon the use of a single IMU, determined the SL through indirect estimation methodologies [16-18].

Zijlstra and Hof (2003) [16] proposed a method for estimating spatial-temporal parameters of gait from trunk accelerations. The IMU was placed on the back at the level of the S2 vertebrae. The method used zero crossing of the forward accelerations for detecting foot contact instances and an inverted pendulum model to estimate the SL. However, in their method, left and right foot contacts identification failed for 6 out of 15 subjects, 12% of the times and the SLs were underestimated in all subjects and at all speeds.

Gonzales and colleagues (2007) [17] proposed a modified version of the pendulum model for taking into account the single stance and double-stance, separately. The IMU was placed on the back at the L3 vertebral level. Traversed distance estimation ranged between 94.5% to 106.7% of the actual traversed distance. No information was provided about the errors associated to the SL estimates.

Shin and Park [18] determined the SL using a linear combination of the walking frequency and the variance of the accelerometric signals recorded by an IMU attached to the subject's waist belt. Experiments were carried out on a single subject. The accuracy of the SL estimation is not reported, but the accuracy of traversed distance resulted to be of about 96%.

The abovementioned methods for the SL estimate are based either on a simplified representation of the human gait mechanics (inverted pendulum model) or on a general linear regressive model. Hence, the SL estimates provided are expected to be affected by the errors intrinsic to the specific model formulation.

Conversely, this study presents a method for the estimation of *rSL *and *lSL *during level walking using a single IMU attached to the pelvis without using gait models. In line with previous approaches, the proposed method requires the identification of the gait events as a preliminary step. To minimize the detrimental drift effects, the IMU acceleration along the DoP is double integrated by means of a combination of direct and reverse integrations [19] of an optimally filtered acceleration.

We hypothesized that the proposed method for the estimation of the SL, would be more accurate than methods found in the literature based on the use of inverted pendulum or regressive models.

The performance of the proposed approach was evaluated on data collected on healthy subjects walking at various speeds, using stereo-photogrammetric (SP) measurements as a gold standard.

## Methods

### Instrumentation

An IMU (FreeSense, Sensorize^®^) featuring a tri-axial accelerometer and two bi-axial gyroscopes (acceleration resolution 0.0096 m/s^2^, angular rate resolution 0.2441 deg/s, unit weight 93 g, unit size 85 mm × 49 mm × 21 mm) sampling data at 100 frames/s was used. A 10-camera BTS^® ^SMART-D stereo-photogrammetric system acquiring at 100 frames/s, (positional accuracy 0.3 mm) was used for validation purposes.

### Step length estimation

**Figure 1:**
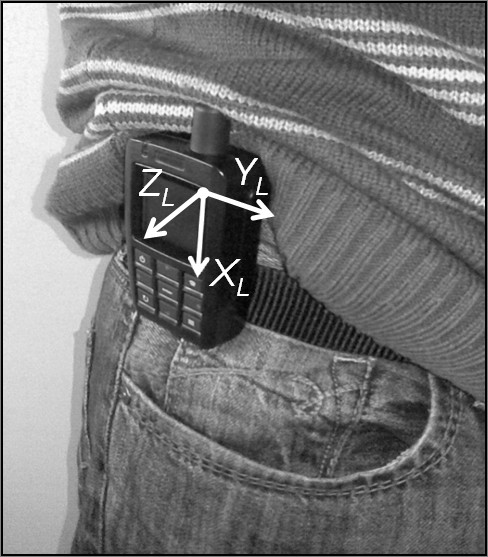
**The IMU location**. The IMU attached to the belt and positioned to the right side of the subject pelvis and relevant LF.

The method described below can be applied to the data obtained from any IMU featuring a tri-axial accelerometer and a three axial gyroscope. The physical quantities, specific forces and angular velocities, provided by each sensors of the IMU, are measured with respect to the axes of a local frame (LF) aligned to the edges of the unit housing. In this application, the IMU was mounted on the right side of the body at the pelvis level with *X_L_*, *Y_L _*and *Z_L _*axes pointing downward, forward, and to the right, respectively (Figure 1).

The method proposed in this paper is based on the assumption that the pelvic displacement along the DoP, between the instants of contra-lateral HS and the first following HS, is equal to the ipsi-lateral step length.

The success of the method relies on the solving of following issues:

a) the identification of gait events;

b) the determination of pelvis displacement along the DoP.

#### Identification of gait events

A gait cycle begins when a foot hits the ground (heel strike - HS) and ends when the next HS of the same foot occurs. When gait cycles can be identified for both sides, then also steps can be identified. A step begins when a contra-lateral HS occurs and ends at the first following HS.

**Figure 2:**
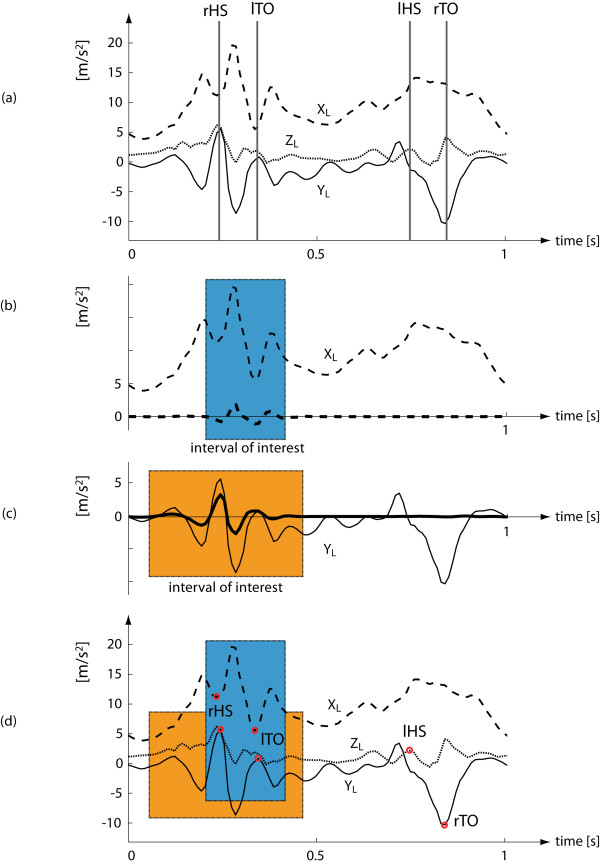
**IMU signals and relevant gait events**. (a) Raw accelerometric signals: *X_L _*pointing downward (dashed line), *Y_L _*pointing forward (solid line) and Z_L _pointing laterally (dot-dashed line). SP-based gait event timings are superimposed (vertical lines). (b) Raw signal on *X_L _*and corresponding reconstructed signal (thick dashed line) used for the definition of the interval of interest. (c) Raw signal on *Y_L _*and corresponding reconstructed signal (thick line) used for the definition of the interval of interest. (d) Circles show the reference points used to estimate gait events from the accelerometric raw signals.

For each recorded gait cycle, the relevant gait events were identified from the IMU signals. By following a heuristic approach [16], a preliminary visual investigation was performed on few samples of data to correlate subject invariant distinctive features in the IMU signals with the relevant gait events extracted from the SP system data (Figure 2a).

A wavelet-based approach was developed to identify intervals of interest where gait event candidates were searched in the accelerometer signals. The *X_L _*and *Y_L _*accelerometer signals were decomposed using a "Stationary wavelet decomposition" [20]. A Daubechies level 5 ("db5") mother wavelet was chosen given its similarity to the IMU signals in the proximity of HS. The original signals were then decomposed in an approximation curve plus ten levels of detail. Thresholds were applied to the first three detail levels and the other detail levels were discarded. Thresholds for these levels were 1/5, 1/4 and 1/3 of its magnitude for the first, second and third level, respectively. The signals were reconstructed using only the first three levels of detail after thresholds were applied. An interval of interest for the accelerometer signals was defined as the interval of time during which the reconstructed signals differed from zero (Figure 2b and Figure 2c). The intervals of interest allow to identify the regions of the signals with localized high frequency components where *rHS *and *lTO *are expected to occur.

Based on the preliminary visual investigation, the right HS (*rHS*) was detected as the instant of time in the middle between the maximum of the *Y_L _*accelerometer signal and the first minimum of the accelerometer signal along the *X_L _*accelerometer signal in the corresponding intervals of interest. The identification of the left HS (*lHS*) required the identification of both right and left toe off instances (*rTO*, *lTO*). The *lTO *was detected as the instant of time in the middle between the first maximum of the *Y_L _*accelerometer signal after the *rHS *and the second minimum of the *X_L _*accelerometer signal in the corresponding intervals of interest. The *rTO *was found as the time of the minimum negative peak value between two consecutive *lTO *and *rHS*. The *lHS *was found as the first local maximum of the *Z_L _*accelerometer signal before the *rTO *(Figure 2d) [21].

#### Estimation of the pelvis displacement along the DoP

The method developed for estimating the pelvis displacement along the DoP requires the knowledge of the gait events and is divided in the following phases:

a) estimation of the IMU acceleration along the DoP;

b) integration of the IMU acceleration along the DoP;

and, when the IMU is located laterally,

c) removal of pelvic rotation contribution to IMU displacement.

#### Estimation of the IMU acceleration along the DoP

To estimate the IMU acceleration along the DoP, the orientation of the LF with respect to the global frame (GF), was estimated using a specifically designed Kalman filter based on both three dimensional acceleration and angular velocity vectors. For a detailed explanation of how the Kalman filter was implemented please refer to Mazzà and colleagues [22].

The GF was defined as follows: the *X_G _*axis coinciding with the direction of gravity, the *Y_G _*axis coinciding with the DoP during level straight walking, and the *Z_G _*axis resulting from the cross product between *X_G _*and *Y_G _*. To define the *Y_G _*axis direction, at the beginning of each acquisition, the IMU *Y_L _*axis was aligned to the DoP of the level straight walking.

The orientation of the LF with respect to the GF at the *i^th ^*instant of time was expressed using the orientation quaternion *^G^***q**_*L*_(*i*).

Let *^L^***f**(*i*) be the vector obtained from the accelerometer signals (expressed in the LF) at the *i^th ^*instant of time, then the acceleration vector *^G^***a**(*i*) expressed in the GF can be computed as:

(1) $${}^{G}a(i){=}^{G}{\left[\begin{array}{c} a_{x}(i)\\ a_{y}(i)\\ a_{z}(i) \end{array}\right]}_{}=\left[\begin{array}{c} -g\\ 0\\ 0 \end{array}\right]{+}^{G}q_{L}{\left(i\right)}^{L}f(i)$$

#### Integration of the IMU acceleration along the DoP

To obtain the velocity and displacement time series along the DoP, an integration technique, the Optimally Filtered Direct and Reverse Integration (OFDRI), and its simplified version, the Optimally Filtered Integration (OFI) [19], was adapted to manage acceleration signals during gait. Both the OFI and the OFDRI were originally designed for step negotiation motor tasks [19] and require the knowledge of the final value of the integral to set a cut off frequency for the high pass filter employed to reduce the effect of the drift in the accelerometer signals. In this application, the cut off frequency was determined from a gait cycle for which the initial and final velocity of the IMU was assumed to be equal. The resulting cut-off frequency was then applied for filtering the acceleration signal along the DoP (*a_x_*(*i*)), one gait cycle at a time. Since the trials started with the subject standing, the initial velocity along the DoP was set to zero. The velocity values found for the final HS were used as initial velocity values for the integration of the following gait cycle.

For gait cycles in which the velocity of the final HS was within a tolerance (± ε, ε = 0.3 m/s) of the initial value then it was forced to be exactly equal to it and the OFDRI was applied. The same updated velocity value was used also as initial velocity of the following gait cycle. The value ε = 0.3 m/s was chosen based on a trial and error approach.

The integration of velocity along the DoP (*v_x_*(*i*)) to obtain displacement, was performed using the OFI. Since, for each cycle, the final value of the integral (the displacement at the final HS) is the unknown quantity, the OFDRI cannot be applied.

Therefore, the resulting estimates of *rSL *and *lSL *of the *j^th ^*gait cycle were:

(2) $$\begin{array}{c} rSL_{j}=\left[s_{x}\left(rHS_{j}\right)-s_{x}\left(lHS_{j}\right)\right]\\ lSL_{j}=\left[s_{x}\left(lHS_{j}\right)-s_{x}\left(rHS_{j}\right)\right] \end{array}$$

where *s_x _*is the displacement along the DoP.

The SL estimation method including the estimation and integration of the IMU acceleration along the DoP was named KOSE (Kalman and Optimal based filtering Step length Estimation).

#### Removal of pelvic rotation contribution to IMU displacement

When the IMU is located on the right side of the pelvis, the IMU displacement along the DoP, at the end of the right step duration (*rT*, the time interval between a *lHS *and the following *rHS*), is larger than the actual *rSL *due to the pelvic angular displacement *θ*, about the vertical axis of the GF, occurring during the same interval of time. Conversely, the IMU displacement at the end of the left step duration (*lT*, the time interval between the *rHS *and the *lHS*) is smaller than the *lSL*. Therefore, applying the equations (2) without taking into account the pelvic rotation, would result in an overestimate of the *rSL *and an underestimate of the *lSL *(vice versa if the IMU is attached to the left side of the pelvis). The amount of the over [under] estimate would be equal to *d*sin(*rθ_j_*) [*d*sin(*lθ_j_*)], where *d *is the inter ASIS (anterior superior iliac spine) distance, and *rθ_j _*[*lθ_j_*] represents the pelvic angular displacement about the vertical axis between the beginning and the end of the *j^th ^*right [left step]. Both *rθ_j _*and *lθ_j _*are obtained from the IMU orientation provided by the Kalman filter.

Therefore, the resulting estimates of *rSL *and *lSL *of the *j^th ^*gait cycle shown in (2) can be corrected as follows:

(3) $$\begin{array}{c} rSL_{j}=\left[s_{x}\left(rHS_{j}\right)-s_{x}\left(lHS_{j}\right)\right]-d\text{sin}\left(r\theta_{j}\right)\\ lSL_{j}=\left[s_{x}\left(lHS_{j}\right)-s_{x}\left(rHS_{j}\right)\right]+d\text{sin}\left(l\theta_{j}\right) \end{array}$$

when the IMU is on the right side of the pelvis and as:

(4) $$\begin{array}{c} rSL_{j}=\left[s_{x}\left(rHS_{j}\right)-s_{x}\left(lHS_{j}\right)\right]+d\text{sin}\left(r\theta_{j}\right)\\ lSL_{j}=\left[s_{x}\left(lHS_{j}\right)-s_{x}\left(rHS_{j}\right)\right]-d\text{sin}\left(l\theta_{j}\right) \end{array}$$

when the IMU is on the left side of the pelvis.

### Experimental session

**Figure 3:**
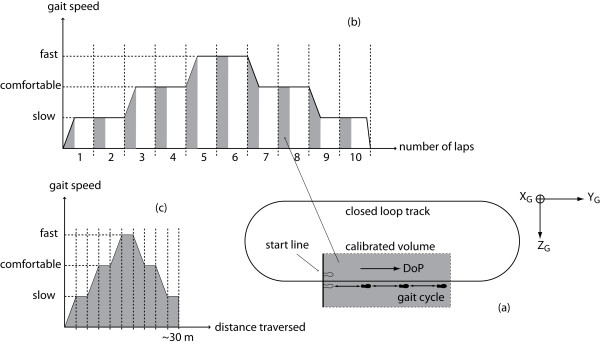
**Experimental data acquisition**. (a) A schematic view of the closed loop track and the calibrated volume (seen from above). (b) Diagram of indicative gait speeds sequence vs. laps of the track. Grey areas represent the portion of the path included in the calibrated volume. (c) Diagram of indicative gait speeds including only portions of the track in the calibrated volume as function of the traversed distance.

Gait data of nine healthy subjects (31 ± 6 yrs) were acquired. Two markers were placed on the right and left heels and toes of each subject. Subjects were asked to walk along a closed loop track of 25 m varying their speed as follows. They started walking from a standing position with their heels aligned to the start line. An adhesive tape, attached to the floor, was used to define the DoP (Figure 3a). For the first two laps they walked at slow speed. At the beginning of the third lap, they increased their speed (comfortable speed) and maintained it for the third and fourth laps. At the beginning of the fifth lap, they further increased their speed (fast speed) and maintained it for the fifth and sixth laps. At the beginning of the seventh lap, they decreased their speed (comfortable speed) and maintained it for the seventh and eighth laps. At the beginning of the ninth lap, they further decreased their speed (slow speed) and maintained it for the ninth and tenth laps. They stopped walking at the end of the tenth lap (Figure 3b).

A 4 m long portion of the walking track was included in the calibrated volume of the SP system, (Figure 3a) with its reference frame made to coincide with the GF. For each lap, SP data of three consecutive gait cycles were recorded.

Only data recorded by the IMU and SP system within the calibrated volume were used for the method validation. Data recorded during each lap were arranged together to obtain a continuous data set formed by 3 × 10 gait cycles (Figure 3c).

### Data analysis

The estimates of the timing of the gait events (rHS, lHS), of the right and left step duration (rT, lT) and of *rSL *and *lSL*, obtained from the heel marker coordinates reconstructed with the SP system were used as gold standard measurements when evaluating the accuracy of the estimates obtained using the IMU [23].

Thus, for each right step *j *and for any given quantity, the differences between IMU estimates and gold standard measurements may be interpreted as estimation error:

(5) $$\begin{array}{c} e_{rHS_{j}}=rHS_{IMU,j}-rHS_{SP,j}\\ e_{rT_{j}}=rT_{IMU,j}-rT_{SP,j}\\ \%e_{rT_{j}}=\frac{rT_{IMU,j}-rT_{SP,j}}{rT_{SP,j}}\\ e_{rSL_{j}}=rSL_{IMU,j}-rSL_{SP,j}\\ \%e_{rSL_{j}}=\frac{rSL_{IMU,j}-rSL_{SP,j}}{rSL_{SP,j}} \end{array}$$

The same processing was applied to the left side.

Descriptive statistics of the above errors were computed.

In addition, to compare our results with previous works, the difference between the IMU estimated distance and the actual traversed distance was computed for each subject.

## Results

### Heel strike detection

All *rHS *and *lHS *instances were successfully detected in all subjects. The mean and standard deviation of errors *e_rHSj _*and *e_lHSj _*are reported in Table 1. On average, the IMU-based estimates of both *rHS *and *lHS *were delayed with respect to the corresponding SP estimates by 0.017 s and 0.027 s, respectively.

**Table 1:** Heel strike detection error

[s]	*e_rHSj_*		*e_lHSj _*	
subject	mean	**s.d**.	mean	s.d
1	0.019	0.027	0.026	0.019
2	0.008	0.021	0.017	0.016
3	0.026	0.017	0.034	0.021
4	0.012	0.007	0.027	0.025
5	0.021	0.031	0.029	0.019
6	0.016	0.013	0.028	0.024
7	0.024	0.022	0.031	0.017
8	0.018	0.02	0.028	0.016
9	0.009	0.019	0.019	0.025

**average**	**0.017**	**0.020**	**0.027**	**0.020**

Mean and standard deviation of errors in detecting right and left heel strikes are reported for each subject. Averaged values across the nine subjects are also reported. All values are in seconds.

### Right and left step duration

The mean and standard deviation of errors *e_rTj _*and *e_lTj _*together with the mean percent errors are reported in Table 2. On average, across subjects, the IMU-based estimates of *rT *were 0.015 s (-2.3%) shorter than the SP measurements. On the contrary, the IMU-based estimates of *lT *were 0.017 s (+2.4%) longer than the SP measurements.

**Table 2:** Step duration error

[s]	*e_rTj _*			*e_lTj _*		
subject	mean	**s.d**.	%	mean	**s.d**.	%
1	-0.021	0.031	-3.10%	0.024	0.025	2.60%
2	-0.015	0.026	-2.80%	0.011	0.017	2.60%
3	-0.014	0.017	-2.10%	0.023	0.019	2.70%
4	-0.009	0.013	-1.70%	0.017	0.017	2.90%
5	-0.014	0.022	-1.90%	0.011	0.023	1.60%
6	-0.011	0.021	-1.50%	0.016	0.019	2.00%
7	-0.022	0.027	-3.20%	0.019	0.025	2.70%
8	-0.017	0.021	-2.50%	0.024	0.025	3.20%
9	-0.01	0.018	-1.50%	0.009	0.018	1.40%

**average**	**-0.015**	**0.022**	**-2.26%**	**0.017**	**0.021**	**2.41%**

Mean and standard deviation of the error (in seconds) and percent error in detecting right and left step duration are reported for each subject. Averaged values across the nine subjects are also reported.

### Right and left SL estimation

Cut-off frequencies employed in both OFI and OFDRI to high-pass filter the original data varied, across the subjects, between the 0.067 Hz and 0.096 Hz. The pelvic rotation about the GF vertical axis was on average across subjects about 5 degrees.

The mean and standard deviation of errors *e_rSLj _*and *e_lSLj _*together with the mean percent errors are reported in Table 3. Percent errors varied across subjects, between -2.6% and +2.9% for the *rSL *and between 0.4% and 2.6% for the *lSL*. On average, across subjects, IMU-based estimates of *rSL *were slightly overestimated by 0.009 m (+1.2%). In contrast, *lSL *were underestimated by 0.008 m (-1.1%).

**Table 3:** Step length error

[m]	*e_rSLj_*			*e_lSLj_*		
subject	mean	**s.d**.	%	mean	**s.d**.	%
1	0.014	0.016	2.0%	-0.010	0.014	-1.9%
2	0.020	0.015	2.9%	-0.017	0.013	-2.6%
3	0.006	0.015	0.8%	0.002	0.016	0.3%
4	-0.001	0.021	-0.2%	0.002	0.019	0.4%
5	0.017	0.018	2.1%	-0.012	0.018	-1.5%
6	0.019	0.019	2.1%	-0.019	0.021	-2.2%
7	0.013	0.015	1.7%	-0.008	0.014	-1.1%
8	-0.008	0.017	-1.2%	0.001	0.019	0.2%
9	0.003	0.013	0.6%	-0.012	0.013	-1.4%

**average**	**0.009**	**0.017**	**1.2%**	**-0.008**	**0.016**	**-1.1%**

Mean and standard deviation of the error (in meters) and percent error in detecting right and left step duration are reported for each subject. Averaged values across the nine subjects are also reported.

### Total traversed distance

The actual and IMU-based estimates of the traversed distance are reported in Table 4. Percent errors over a traversed distance of about 30 m, varied across subjects, between -0.54 m (-1.6%) and 0.54 m (+1.7%). The average estimate of the traversed distance is equal to 0.1%.

**Table 4:** Total traversed distance

subject [no.]	traversed distance [m]	estimated distance [m]	difference [m]	difference [%]
1	32.65	33.02	0.37	1.1
2	30.99	31.46	0.46	1.5
3	31.17	30.80	-0.37	-1.2
4	31.71	32.25	0.54	1.7
5	30.37	29.91	-0.46	-1.5
6	33.44	32.89	-0.54	-1.6
7	31.40	31.74	0.34	1.1
8	32.11	31.78	-0.33	-1.0
9	30.63	31.00	0.37	1.2

**average**	**31.61**	**31.65**	**0.04**	**0.1**

Actual and estimated traversed distance (in meters) obtained from the SP and IMU data respectively, along with the difference (in meters) and percentage difference. Averaged values across the nine subjects are also reported.

## Discussion and Conclusion

A method for determining both right and left SL during level walking using a single IMU to be used either indoor or outdoor, without space limitations and for prolonged periods of time, was presented and validated.

SL estimates were obtained directly from the IMU displacement between two consecutive HS, by employing an original method (the KOSE method) which double integrates the IMU acceleration along the DoP obtained after applying a Kalman filter. The KOSE method includes an adaptation to gait of an optimal integration technique originally proposed by Zok and colleagues [19] to reduce the errors caused by the drift affecting the dynamometric signals.

The method was tested on healthy subjects while they were walking increasing and decreasing their speed, five times along 30 m. The algorithm for the identification of left and right HS never failed in all the subjects analyzed. Both right and left HS were detected with an average time delay corresponding to 1.7 samples and 2.7 samples, respectively (sample frequency 100 frames/s). These errors implied that the right step duration was on average two samples shorter than the left one. Despite the shorter estimated step duration, the *rSL *resulted, over the different subjects, overestimated by 1.2%. The maximum mean error in estimating the SL was overestimated by 2.9% for the *rSL *and underestimated by 2.6% for the *lSL*. The main explanation for the side to side differences, is probably that the effect of the pelvic rotation, associated to the asymmetrical IMU positioning, was not completely compensated for by the correction term *d*sin(*θ_j_*). In fact, in this regard, several assumptions were not completely satisfied: being attached to the belt of the subject, the IMU was not rigidly moving with the pelvis and *d*, which is taken as the inter-ASIS distance may differ from the distance between the LF origin to the pelvis vertical axis of rotation. As expected, the maximum percent error on the total traversed distance was to 1.7% and therefore smaller than the maximum SL percent error. It is worth noticing that the estimate of the traversed distance was never consistently either an overestimate or an underestimate (average over subjects equal to 0.1%).

Several methods to estimate *rSL *and *lSL*, using a single IMU, have been proposed in the literature [16-18,24]. A common feature of these methods is that the SL estimates were derived using indirect methodologies, either based on inverted pendulum models for level human walking representation [25,26] or based on general regressive equation in which SL is expressed as function of the step frequency and accelerometric signal variations. Unfortunately, no data on the accuracy of the *rSL *and *lSL *estimates were provided in abovementioned studies. Therefore, a straightforward comparison among methods is not possible. However, Zijlstra and Hof (2003) [16] reported a consistent SL underestimation. As a consequence, also the mean speed (mean SL divided mean step time) was underestimated and to correct it, they introduced a coefficient of 1.25 heuristically determined. It could then be inferred that the SL estimations were affected by errors of about 20-30%. In Gonzales and colleagues (2007) [17], the performance of their method was evaluated by assessing the errors of the distance traversed in each acquisition. Across subjects, errors ranged from -6.5% to +6.0%. Shin and Park [18] tested their method on a single subject walking at a constant speed for a 70 m straight trajectory. Three data set were recorded (slow, normal and fast). Across trials, errors for the traversed distance varied between 1% to 3% with an accuracy error of 3.7% in the worst case.

In our study the errors in estimating the traversed distance ranged from -1.6% to +1.7% across subjects.

The smaller estimation errors compared to previously publish methods, confirm our initial hypothesis that the KOSE method improves the accuracy with respect to indirect methods based on the use of general models, which may not always take into account subject specificity in gait.

In this study, the IMU was attached to the subject's belt on the right side. This location was chosen to reduce subject discomfort and because it is a practical solution for monitoring daily life motor activities. However, it can be hypothesized that attaching the IMU centrally in the back (S2 or L3 vertebral level) similarly to Zijlstra and Hof (2003) [16], Gonzales and colleagues (2007) [17] and Shin and Park [18], the residual errors related to the pelvic rotation correction would be reduced and it would not be necessary to correct for the pelvic rotation effects.

In this study we presented results relative to gait event timings and SL estimations. Additional gait parameters can be easily calculated from the estimated parameters. Therefore, using a single IMU, we were able to provide excellent estimates of both temporal and spatial gait parameters bilaterally.

It is important to stress that the KOSE method was tested on experimental conditions fairly similar to those which can be encountered in the real life: different gait speeds (three gait speed levels), numerous velocity transients (five velocity transients on 30 m). It is reasonable to expect that, in healthy subjects, errors in traversed distance estimation during daily monitoring activity would be of the same order of magnitude of those presented.

The data set, derived from the experiments carried out in the present study, referred to a straight level walking. For this specific method validation, the GF was therefore defined, at the beginning of each acquisition with the *Y_G _*axis coinciding with the DoP. The IMU acceleration component along the DoP, was then calculated by projecting the accelerometric signal, after having removed the gravitational contribution, on the GF. In general, if during the acquisition, the subject varies the DoP more than once, it would be necessary to define for every different DoP, an auxiliary GF with the *Y_G _*axis aligned to the relevant DoP. However, the solution of the latter issue is out of the scope of the present study.

There are some limitations to the current proof of concept which need to be addressed before using the presented methodology to estimate gait spatial and temporal parameters in clinical contexts. One issue is related to the algorithm used to identify the gait events, which is normally based on IMU signal features. Such features may change or even disappear in pathologic subjects. Another issue is related to the assumption that the pelvis displacement along the DoP between HS equals the SL. It is certainly true in a symmetric gait, but in some gait disorders the pelvis displacement may not be in phase with the feet displacement as much as in healthy subjects. Future studies need to be performed to test the method performance on pathological gait [27].

In conclusion, the method proposed allows for an accurate estimate of right and left step length using a minimal invasive experimental set up through an optimal integration procedure of the accelerometric signals.

## List of abbreviations

DoP: direction of progression; GF: global reference frame; HS: heel strike; IMU: inertial measurement unit; LF: local reference frame; lHS: left heel strike instant; lSL: right step length; lTO: left toe off instant; OFDRI: optimally filtered direct and reverse integration; OFI: optimally filtered integration; rHS: right heel strike instant; rSL: right step length; rTO: right toe off instant; SL: step length; SP: stereo-photogrammetric.

## Competing interests

The authors declare that they have no competing interests.

## Authors' contributions

AK, AC, and UD participated in the conceptualization of the methods proposed and contributed to the definition of the experimental design and the data analysis. AK was involved in the methods development, acquisition and the analysis of the data. All authors revised and approved the current version of the manuscript.
